# Impact of menopause hormone therapy, exercise, and their combination on bone mineral density and mental wellbeing in menopausal women: a scoping review

**DOI:** 10.3389/frph.2025.1542746

**Published:** 2025-05-12

**Authors:** Olivia Platt, James Bateman, Shagaf Bakour

**Affiliations:** ^1^ Sandwell and West Birmingham NHS Trust, West Bromwich, United Kingdom; ^2^ School of Health Sciences, College of Medicine and Health, University of Birmingham, Birmingham, United Kingdom; ^3^ Royal Wolverhampton NHS Trust, Wolverhampton, United Kingdom; ^4^ Institute of Clinical Sciences, University of Birmingham, Birmingham, United Kingdom; ^5^ Aston Medical School, Aston University, Birmingham, United Kingdom

**Keywords:** menopause, menopause hormone therapy, exercise, bone mineral density, osteoporosis

## Abstract

**Background:**

Osteoporosis, a condition marked by low bone mineral density (BMD) and structural deterioration, affects more women than men over 50 globally. In women, declining estrogen during the menopause accelerates bone resorption, heightening fracture risk. An association between osteoporosis and depression, frailty fractures and poor quality of life has been identified. Both menopause hormone therapy (MHT) and exercise are shown to improve BMD, with MHT reducing bone resorption and exercise promoting bone formation. This review examines the effectiveness of MHT, exercise, and their combination in managing menopausal osteoporosis.

**Method:**

A multifactor scoping review was conducted to address osteoporosis and MHT, osteoporosis and exercise, and osteoporosis and MHT and exercise combined.

**Results:**

Initial searches identified 15,158 studies, narrowed to 20 meeting the inclusion criteria. MHT and exercise are effective in preserving BMD in menopausal women. Combined estrogen and progesterone MHT is more effective than estrogen-only, with studies suggesting that MHT prescribed at low doses for longer durations more effectively preserves BMD. Resistance training (RT) completed 2–3 days per week at a moderate-to-high intensity combined with impact activity completed at a minimum of 3 days per week is optimal for improving BMD in menopausal women, while low-impact exercises provide supplemental benefits. Combining MHT with exercise enhances BMD more than either alone.

**Conclusion:**

This review highlights that combining MHT and structured exercise is most effective for enhancing BMD in menopausal women. Given certain safety considerations surrounding MHT in some women, exercise remains a cornerstone for the prevention and management of osteoporosis as well as for promoting overall wellness.

## Introduction

Osteoporosis is a disease characterized by bone structural deterioration and low bone mineral density (BMD), measured by dual-energy x-ray absorptiometry (DXA), to produce a T-score (1). BMD is categorized as normal when the T-score is greater than −1, osteopenia when it falls between −1 and −2.5, and osteoporosis when it is less than −2.5. Osteoporosis affects one in three women and one in five men over the age of 50 worldwide (2), with osteoporosis in women increasing from 6.8% at ages 50–59 to 25.7% at ages 70–79 and 34.9% in those over 80 (3). Bone remodeling occurs throughout life to maintain the strength and integrity of bone. This is achieved through osteoblasts, bone-forming cells that deposit new bone tissue, and osteoclasts, bone-resorbing cells that break down bone tissue. Estrogen plays an important role in the complex interplay of new bone formation and bone resorption. It enhances osteoblast activity, reducing osteocyte apoptosis (recruitment of osteoclasts to initiate bone resorption) and promoting osteoclast apoptosis (osteoclast cell death) to inhibit osteoclast function, suppressing bone resorption (4).

The menopause is a natural biological process marking the end of a woman’s reproductive years, in which the loss of ovarian follicular function results in decreased estrogen and progesterone. The perimenopause typically starts around the age of 45, whereby estrogen levels begin to fall, characterized by irregular periods and menopause symptoms. Menopause is then defined as 12 consecutive months without a menstrual period, with the following phase termed postmenopause. Therefore, as estrogen levels reduce during the menopausal transition, there is greater bone resorption than bone formation, resulting in decreased BMD and risk of osteoporotic fractures (5).

Menopause hormone therapy (MHT) can reduce excessive bone resorption by inhibiting osteoclast activity (6). The two main types of MHT are combined MHT and estrogen-only MHT (7). Combined MHT includes both estrogen and progestogen and is recommended for women with an intact uterus to protect the endometrium from the unopposed effects of estrogen (7). Estrogen-only MHT is suitable for women who have undergone hysterectomy, as there is no endometrium to protect.

Endogenous estrogens produced by the human body include estradiol, estrone, and estriol. In MHT, estrogen may be administered in the form of conjugated equine estrogens (CEOs) or synthetic bioidentical preparations that are structurally identical to these endogenous hormones. MHT may also include phytoestrogens—plant-derived compounds from sources such as soy or yams—which have a molecular structure different from human estrogens. While phytoestrogens can bind to estrogen receptors and exert mild estrogenic effects, they are not bioidentical and do not replicate the full biological activity of endogenous estrogens.

CEOs, derived from the urine of pregnant mares, contain a mixture of estrogen compounds such as estrone sulfate and equilin sulfate, which are metabolized into active estrogens in the body (8). CEOs have been widely used in MHT for the management of menopausal symptoms (9). However, their use has declined following the Women’s Health Initiative (WHI), which reported an increased risk of adverse outcomes, including breast cancer, thromboembolism, cardiovascular disease, and endometrial cancer (10).

Progestogens are an essential component of combined MHT to counteract the proliferative effects of estrogen on the endometrium. These are available in two main forms: synthetic and natural. Synthetic progestogens, also known as progestins, include medroxyprogesterone acetate (MPA), norethisterone, levonorgestrel, norgestrel, drospirenone, and dydrogesterone. MPA has been widely used in combined MHT but is associated with an increased risk of breast cancer (11), prompting a shift toward alternative progestogens with potentially better safety profiles.

Natural progestogens include micronized progesterone (e.g., Utrogestan), which is plant-derived and bioidentical to endogenous progesterone. This offers a more physiological and potentially more tolerable option for MHT.

Another form of MHT is Duavive®, which combines conjugated estrogens with bazedoxifene acetate, a selective estrogen receptor modulator (SERM) (12). Duavive is typically prescribed for postmenopausal women with a uterus when progestogen therapy is unsuitable or unnecessary. Bazedoxifene provides endometrial protection, eliminating the need for progestogen (13).

Mechanical loading applied during exercise leads to an osteogenic response, stimulating bone growth and increased BMD, through the mechanosensory role of osteocytes. When osteocytes receive signals of mechanical load, the subsequent mechanotransduction, biochemical and intracellular changes in response to mechanical stimuli, impact the function of osteoblasts and osteoclasts to modify homeostasis (14). Exercise prescriptions vary by type and intensity, including resistance training (RT), aerobic and impact training, and Tai Chi. RT intensity is measured as a percentage of one-repetition max (1RM), defined as the maximum amount of weight lifted for one repetition, while aerobic intensity uses a percentage of maximum heart rate (MHR), which is calculated as 220 minus age. Exercise intensity is often categorized based on percentages of MHR or perceived exertion. The American Heart Association (AHA) (15) defines moderate-intensity exercise as 50%–70% of MHR and vigorous-intensity exercise as 70%–85% of MHR.

In summary, bone remodeling is maintained by a balance between osteoclastic resorption and osteoblastic formation. In menopause, reduced estrogen levels disrupt this equilibrium—enhancing resorption through increased receptor activator of nuclear factor kappa-β ligand (RANKL) and decreased osteoprotegerin—contributing to osteoporosis. Regular exercise promotes osteoblastic activity and bone strength, while hormonal therapy can mitigate the effects of estrogen deficiency, highlighting the critical interplay between mechanical loading and hormonal regulation in maintaining bone health.

Research demonstrates the impact of the menopausal transition on women’s mental health and quality of life (16). Bromberger et al. (17) identified that peri-menopausal women and early postmenopausal women (PMW) are two to four times more likely to experience a significant depressive episode. Due to physiological changes such as weight gain and muscle loss during the menopause transition, women often experience low self-efficacy and body dissatisfaction (18). Further to this, growing evidence highlights the bidirectional relationship between postmenopausal osteoporosis and mental health disorders (19). A systematic review and meta-analysis by Wang et al. (20) found that PMW with osteoporosis are significantly more likely to experience depressive symptoms compared to those without osteoporosis. In addition, research by Smith et al. (21) demonstrated that chronic arthralgia, common among osteoporotic 121 patients, is associated with poorer mood, lower quality of life, and heightened depression scores.

Osteoporosis-related frailty fractures have a profound impact on health-related quality of life (HRQoL), leading to chronic pain, reduced mobility, loss of independence, and an increased healthcare burden (22). These factors contribute to social isolation and emotional distress, further exacerbating psychological symptoms in PMW. Research has consistently demonstrated a strong association between low BMD, osteoporotic fractures, and a decline in self-reported mental health (23, 24). Alarmingly, osteoporosis-related physical limitations have been linked to higher rates of suicidal ideation in PMW, highlighting the urgent need for effective management strategies (25). The psychological impact of osteoporosis is likely driven by a combination of biological, psychological, and social factors, including chronic pain, reduced mobility, and diminished quality of life.

MHT has been widely recognized as an effective intervention for mitigating BMD loss. Beyond its skeletal benefits, MHT may also contribute to improved mental wellbeing, as stabilizing estrogen levels has been shown to slow bone resorption while positively influencing mood regulation and cognitive function (26). Similarly, exercise plays a critical role in osteoporosis management, not only by enhancing BMD but also by modulating the hypothalamic–pituitary–adrenal (HPA) axis, leading to reduced cortisol secretion (27). Since elevated cortisol levels are associated with sleep disturbances, anxiety, and mood fluctuations (62), exercise may offer both physical and psychological benefits for osteoporotic PMW. Moreover, a previous scoping review has highlighted the significant impact of regular exercise on the overall quality of life in this population (28).

These findings emphasize the need for a holistic management approach in postmenopausal osteoporosis, addressing both physical symptoms and psychological wellbeing. Given the consequences of osteoporosis on physical and mental health, it is essential to establish the most effective management strategies to preserve BMD and prevent osteoporosis in menopausal women. While both MHT and exercise independently show promise in maintaining bone density, their combined effects remain less explored. This review focuses on evaluating the direct impact of these interventions on BMD, as identifying the most effective strategy for bone health is a crucial first step before considering secondary outcomes such as psychological wellbeing. Although mental health is undeniably linked to osteoporosis, it was not included in this review, as the primary aim was to assess the physiological effects of MHT and exercise on BMD. Therefore, this scoping review aims to evaluate the effectiveness of MHT alone, exercise alone, and their combination in improving BMD in menopausal women, providing valuable insights into optimizing treatment approaches for this population.

## 2 Methods

The Arksey and O’Malley (29) framework was used to conduct this scoping review, with the PRISMA-ScR checklist to report the results. The research question guiding this systematic review is: What is the impact of MHT alone, exercise alone, and their combination compared to no intervention or a placebo on BMD in menopausal women? Addressing multiple interventions posed challenges in evaluating outcomes beyond BMD. While our initial intent was to assess secondary outcomes, such as the impact on mental health, we focused on BMD to maintain methodological rigor and raise awareness of the significant impact on mental health. This decision highlights the need for further research into the under-investigated area of these interventions’ effects on mental health, suggesting a dedicated systematic review on this topic. To define the research question and guide the study selection process, we used the PICO framework. The population was menopausal women, including both perimenopausal and postmenopausal women; the intervention involved MHT alone, exercise alone, and MHT and exercise combined; the comparison included no intervention or placebo; and the outcome assessed improvements in BMD measured by DXA scans, shown as a T-score. Given the research question, a multifactor search was required to address osteoporosis and MHT, osteoporosis and exercise, and osteoporosis and MHT and exercise combined.

A comprehensive search was performed on Embase, EMCARE, MEDLINE, CINHAL, and the Cochrane Library Database of Systematic Reviews. Due to the large volume of studies generated from the initial search, a second, more refined search was carried out. Only records that met the following criteria were included: English language, published between 2004 and 2024; and study types including randomized control trials (RCTs), cohort studies, case-control studies, systematic reviews, meta-analyses, cross-sectional studies, or qualitative studies.

The initial search on osteoporosis and exercise yielded a substantial number of studies. As a result, the search was further narrowed to include only systematic reviews and meta-analyses, or systematic reviews that included meta-analyses. Studies were then exported into RefWorks®, where titles and abstracts were screened. The inclusion and exclusion criteria were applied to identify the most relevant studies (Table 1).

**Table 1:** Eligibility criteria.

Inclusion	Exclusion
General criteria	
Population: Peri-menopausal and postmenopausal women	Pre-menopausal women defined as those not experiencing menopausal symptoms and/or irregular bleeding, and women with premature ovarian insufficiency (POI).
Outcomes: Bone mineral density (BMD)	Studies that focus on other osteoporosis treatments (e.g., dietary supplements, calcium, vitamin D) as the primary intervention.
Measure: Dual-energy x-ray absorptiometry (DXA) scan or fracture risk	Studies not published in English or published before 2004.
Language: English	Animal studies
Time frame: Published between 2004 and 2024	Studies that do not use DXA scans to measure BMD or fracture risk as primary outcome measures
Osteoporosis and MHT	
Intervention: Studies focusing on MHT (any type: estrogen, combined estrogen-progesterone) as a sole treatment for managing BMD osteoporosis	Studies with interventions unrelated to osteoporosis BMD management (e.g., MHT for cardiovascular outcomes only)
Study design: Randomized controlled trials (RCTs), controlled clinical trials, cohort studies, systematic reviews, and meta-analyses	Not a RCT, controlled clinical trial, cohort study, systematic review, or meta-analysis
Osteoporosis and exercise	
Intervention: Studies evaluating the effect of exercise (weight-bearing, resistance, balance training, aerobic exercise, low impact, and high impact) as a sole intervention for BMD osteoporosis	Not a systematic review, observational study, case study, or single-arm trial on exercise
Study design: Systematic reviews and meta-analyses only (due to a large number of studies on exercise and osteoporosis)	
Osteoporosis and MHT and exercise	
Intervention: Studies that evaluate the combined impact of MHT and exercise on osteoporosis management	Studies that do not assess the combined effect of both MHT and exercise
Study design: RCTs, controlled clinical trials, cohort studies, systematic reviews, and meta-analyses	Not a RCT, controlled clinical trial, cohort study, systematic review, or meta-analysis

The search terms were adjusted to align with the indexing systems of each database. In EMCARE and MEDLINE, Medical Subject Headings (MeSH) and keyword variations were used, including: [“Osteoporosis” OR “Bone density” (MeSH)] AND [“Menopause” OR “Postmenopause” OR “Perimenopause” (MeSH) OR “Post-menopause” OR “Peri-menopause” OR “Menopause*” (Keywords)] AND [“Hormone replacement therapy” OR “Estrogen Replacement Therapy” (MeSH) OR “HRT” (Keyword)] OR [“Exercise” (MeSH) OR “Physical activity” (Keyword)].

In CINAHL, the equivalent CINAHL Subject Headings were applied along with keywords to capture relevant studies, such as: [“Osteoporosis” OR “Bone Density” (CINAHL heading)] AND [“Menopause” OR “Postmenopause” OR “Perimenopause” (CINAHL headings) OR “Post-menopause” OR “Peri-menopause” OR “Menopaus*” (Keywords)] AND [“Hormone replacement therapy” (CINAHL headings) OR “HRT” OR “oestrogen replacement therapy” (Keywords)] OR [“Exercise” OR “Physical Activity” (CINAHL headings)].

The Cochrane Library Database of Systematic Reviews was searched using broad keyword variations without MeSH terms to maximize retrieval, ensuring coverage of systematic reviews related to osteoporosis management in menopausal women. Boolean operators and truncation were consistently used across all databases to refine the search and capture relevant literature. Key terms used across the databases included: (“Osteoporosis” OR “Bone density”) AND (“Postmenopause” OR “Post-menopause” OR “Perimenopause” OR “Menopause*”) AND (“Hormone replacement therapy” OR “HRT” OR “oestrogen replacement therapy”) AND (“Exercise” OR “Physical activity”).

Relevant information from the selected studies was documented in a Microsoft Word document under the following headings: author(s), year of publication, study design, study aim, intervention, and key findings. The data were then analyzed and synthesized to align with the research findings.

## 3 Results

### 3.1 Study selection

**Figure 1:**
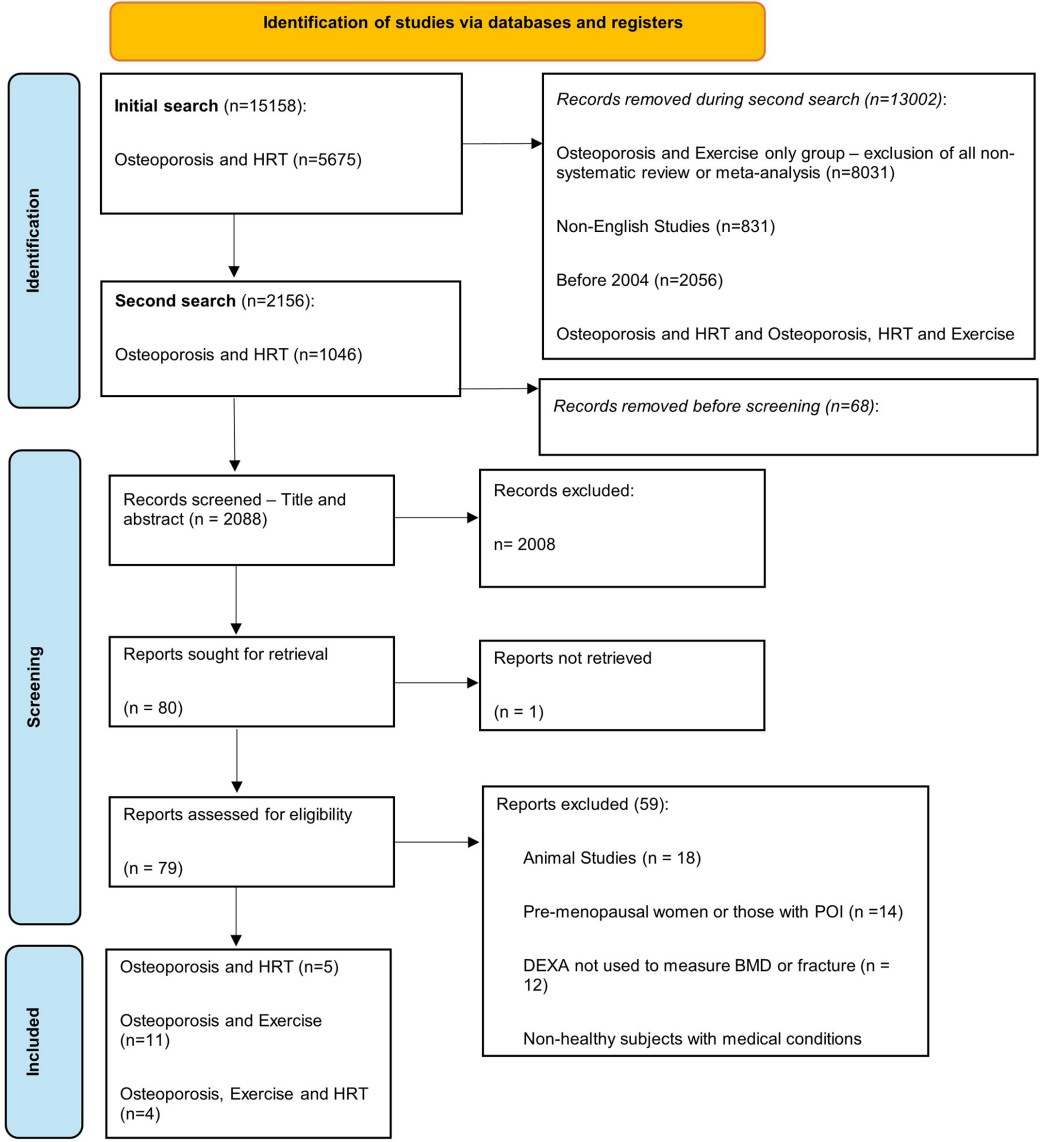
PRISMA flowchart for the identification of the studies.

A total of 15,158 studies were identified for osteoporosis and MHT (*n* = 5,675), osteoporosis and exercise (*n* = 8,551), and osteoporosis and MHT and exercise combined (*n* = 932). The second circumscribed search identified 2,156 studies, of which 68 were removed due to being duplicates. Moreover, 2,008 studies were excluded after title and abstract screening, with 80 sought for retrieval. Furthermore, 79 studies were assessed for eligibility, with 59 excluded according to our eligibility criteria. Thus, 20 studies met the inclusion criteria and were included in this review. There were five studies for osteoporosis and MHT, 11 studies for osteoporosis and exercise, and four studies for osteoporosis and MHT and exercise combined (Figure 1).

### 3.2 Study characteristics

Of the 20 studies, the study designs included cohort studies (*n* = 1), case-control studies (*n* = 1), RCTs (*n* = 4), systematic reviews (*n* = 2), meta-analyses (*n* = 3), and systematic reviews with meta-analyses (*n* = 9). DXA was used in all studies, with one study also measuring the incidence of hip fractures. Among sites measured, 17 assessed lumbar spine (LS) BMD, 17 measured femoral neck (FN) BMD, one study measured the greater trochanter (GT), and one study did not specify the site of measurement. The study characteristics and results are described in detail in Table 2 and summarized in the following sections.

**Table 2:** Study characteristics and findings.

Authors Study design	Study aim	Population	Intervention	Outcome measures of interest	Summary of key findings
MHT and osteoporosis					
Moilanen et al. (30) Longitudinal cohort study	To examine the characteristics of 25-year changes in femoral BMD after menopause.	3,222 women *Age:* mean age 53.3 years (range, 47–59 years) at baseline *Menopause status:* PMW—62.1% (*n* = 2,002) of women already PMW	The study population was divided into bone loss quartiles (FQ1–FQ4); FQ1 had the highest bone loss rate and FQ4 had the lowest rate. MHT use was self-reported during the 25 years. *MHT type*: NR *MHT dosage*: NR *MHT duration:* FQ1 45.2, FQ2 54.8, FQ3 71.5, and FQ4 91.9 months	DXA scan of the FN every 5 years from baseline	There was a higher prevalence of MHT use in the group with the lowest BMD loss Prevalence of self-reported MHT use was observed in 26.3% of the women in the lowest bone loss quartile (FQ1), 32% in FQ2, 35.5% in FQ3, and for 41.5% of women in FQ4 (*p* = 0.002 between FQ1 and FQ4) The duration of MHT during the 25-year follow-up was 65.8 months on average. The duration in months of MHT in the bone loss quartiles increased from FQ1 (45.2), FQ2 (54.8), and FQ3 (71.5) to FQ4 (91.9)
Heikkinen et al. (31) A long-term, single-center, randomized study	To investigate the effect of low-, moderate-, and high-dose continuous-combined hormone replacement (ccMHT) therapy and its discontinuation on bone in PMW	279 women. Mean age at baseline: 56.2 Mean age at menopause: 49.0 *Menopause status:* PMW	*MHT type*: Estradiol valerate and medroxyprogesterone acetate (MPA) MHT *dosage:* Low dose—1 mg E2 V þ 2.5 mg MPA (1 þ 2.5); moderate dose—1 mg E2 V þ 5 mg MPA (1 þ 5); high dose—2 mg E2Vþ5 mg MPA (2 þ 5); all individuals were treated with the lowest dosage (1 þ 2.5) for the final 6 months *MHT duration:* 9 years;. discontinuation from year 9 to 10	DXA scan of the FN and LS, measured at baseline, at 6 and 12 months and annually for 9 years	LS BMD increased progressively in all treatment groups over 9 years (*P* < 0.0001) vs. baseline. FN BMD increased from baseline to 5–6 years From year 9 to 10 after discontinuation of ccMHT, 98 women had BMD loss ≥2% and 58 had accelerated (≥4%) loss of LS BMD. Accelerated bone loss was greater after discontinuation of the higher dosages of ccMHT. LS BMD in year 10 remained above the baseline
Manson et al. (32) Randomized controlled trial	To provide an overview of findings from the two Women’s Health Initiative (WHI) hormone therapy (HT) trials with post-intervention follow-up	27,347 women, 16,608 women with a uterus, and 10,739 women with hysterectomies Age: 50–79 years *Menopausal status:* PMW	*MHT type:* Women with uterus—oral CEE, CEE plus MPA, and placebo Hysterectomized women—oral CEE or placebo *MHT duration:* NR	Hip fracture	Women in the CEE + MPA and CEE groups, compared to placebo, had statistically significant 33% reductions in hip fracture A significant fracture benefit persisted at 13 years for CEE + MPA
Prior et al. (33) A systematic review and meta-analysis of randomized controlled trials	To assess whether there is a difference in LS BMD in which menopausal women were directly randomized to the same dose of estrogen alone (ET) or with progesterone (EPT)	*Studies included:* five studies Dates of studies: 1996–2005 *Participants:* study sizes ranged from 24 to 337 *Mean age:* 51–56 years old *Menopausal status:* PMW, 1–12.8 years postmenopause	*MHT type:* primarily CEE, one trial administered 1 mg oral 17β-estradiol/d (E_2_). The progesterone/progest was MPA *MHT dosage:* CEE—0.625, 0.45, or 0.3 mg/day; 17βE_2_—1 µg; MPA—1.25–20 mg, with 2.5 mg/day being the most common dose *MHT duration:* NR	DXA LS, TH, and FN BDM	Within-study EPT arms compared with the ET ones showed a significantly greater mean difference in LS BMD of +0.68% change/year (*P* = 0.0001)
Ran et al. (34) Double-blind, randomized, parallel placebo-controlled study	To observe the effectiveness and safety of MHT to prevent bone loss in Chinese women during the menopausal transition and early menopause	*Participants:* 124 in the menopausal transition group; 96 early PMW Women were randomly assigned to MHT and placebo groups	*MHT type*: Estradiol valerate and MPA, blank starch tablets were used as the placebo *MHT dosage:* NR *MHT duration:* NR	DXA scan of the LS and FN at 0, 12, 24, 36, 48, and 60 months	*Early menopause group*: in years 1–2 of follow-up, the MHT group showed a significant increase in the LS BMD (*p* < 0.01 vs. baseline) and a further decrease from year 2 onwards, though the BMD was still higher than at baseline. In contrast, the placebo group showed a significant decrease in LS BMD (*p* < 0.05 vs. baseline) over 5 years *Menopause transition group*: After 1 year of treatment, the MHT group showed a significant increase in the LS BMD (*p* < 0.01 vs. baseline). The values tended to decrease slightly in the FN in year 2
Osteoporosis and exercise					
Hejazi et al. (35) A systematic review with meta-analysis of RCTs	To examine the efficacy of exercise training regimens for changing BMD in older PMW	*Studies included:* 53 RCTs *Participants:* 2,896; 1,613 in the intervention groups and 1,253 in the control groups *Mean age:* 60–82 *Menopause status:* PMW	*Exercise type:* RT, aerobic training, walking, weight-bearing training, whole body vibration, water-based RT and fitness, and Tai Chi *Frequency:* 2–5 sessions/week *Intervention duration:* 4 months–2 years *Session duration:* 10–90 min	DXA scan of FN BMD and LS BMD	Exercise significantly increased FN BMD (*P* = 0.0001). RT combined (aerobic + RT) and Tai Chi training significantly increased FN BMD; aerobic, whole-body vibration, walking, and weight-bearing training did not significantly increase FN BMD Exercise significantly increased LS BMD (*P* = 0.0001). Aerobic training and combined (aerobic + RT) significantly increased LS BMD; resistance training, whole-body vibration, walking, Tai Chi, and weightbearing training did not significantly increase LS BMD Exercise training did not change total hip BMD
Kemmler et al. (36) A systematic review and meta-analysis	To explore the effects of (dynamic) resistance exercise (DRT), weight bearing (WB) exercise, and combined WB&DRT on BMD in PMW	*Studies included:* 74, WB *n* = 30, DRT *n* = 18, and WB&DRT interventions *n* = 36 *Participants:* 2,793 in the exercise group and 2,319 in the control group *Mean age:* 51 ± 2 years and 77 ± 3 years *Menopause status:* PMW	*DRT: Exercise type:* Full body unilateral exercise and compound exercises using machines and free weights *Frequency:* Three sessions/week *Session duration:* 1–120 min *Intensity: 30*%–80% 1RM *Intervention duration:* 6–24 months *WB: Exercise type*—walking with additional load, walking/running, Tai Chi, jumping or rope skipping, heel drops, stepping, standing on one leg, and combined weight bearing types (e.g., heel drops, jumping skipping; stairclimbing) *Frequency*: 2–10 sessions/week. *Session duration:* 20–50 min. *Intensity:* 40%–80% HRMax. *Intervention duration:* 6–30 months. *Combined WB and DRT:* Exercises: walking, running, stepping, movement games, dancing, and a DRT on machines or with free weights *Frequency:* < 2–8 sessions/week*SessionSession duration:* 40–100 min *Intensity:* 60%–90% 1RM *Intervention duration:* 6–26 months	DXA scan of FN, LS and TH	LS-BMD improved significantly for DRT (*P* = 0.009), WB exercise (*P* = 0.037), and combined WB&DRT exercise (*P* = 0.001). No significant differences between the types of exercise were observed (*P* = 0.508). All types of exercise revealed a similarly high level of heterogeneity between their trials (*I*^2^ = 76.3–76.5%) FN-BMD improved significantly for DRT (*P* < 0.003), WB exercise (*P* = 0.004), and combined WB&DRT exercise (*P* = 0.001) TH-BMD improved significantly for DRT (*P* = 0.001), WB exercise (*P* < 0.001), and combined WB&DRT exercise (*P* < 0.001) No significant differences between the types of exercise were observed for the LS, FN, or TH
Kistlet-Fischbacher et al. (37) Meta-analysis	To determine the effect of low, moderate, and high intensity exercise on BMD at the LS, FN, and TH. To determine the effect of different types of exercise, performed at low, moderate, and high intensity on LS, FN. and TH BMD	*Studies included:* 53 *Participants:* 3,941, 1,948 allocated to exercise and 1,582 to control *Mean age:* Between 51.4 and 79.3 years. *Menopause status:* PMW	Low intensity exercise: *Exercise type:* Walking, Pilates, Thai Chi, and swimming *Frequency:* 2–7 sessions week *Session duration:* 40–60 min *Intensity:* RT ≤65% 1RM with >16 repetitions, GRF < 2× bodyweight *Intervention duration:* 6–24 months Moderate intensity exercise: *Exercise type:* RT, aerobic and impact exercise *Frequency:* 3–6 sessions week *Session duration:* 10–60 min *Intensity:* RT 65%–80% 1RM with 8–15 repetitions and GRFs of 2–4× bodyweight *Intervention duration:* 6–30 months High-intensity Exercise: *Exercise type:* RT, aerobic and high-intensity impact exercise *Frequency:* 2–3 sessions week *Session duration:* 30–60 min *Intensity:* ≥ 80% 1RM with <8 repetitions and GRF >4 times bodyweight *Intervention duration:* 6–12 months	DXA scan of FN, LS and TH	LS BMD: Significant positive effect of exercise on LS BMD (*P* < 0.001). Greater effects for high intensity than moderate and low intensity FN BMD: Significant positive effect of exercise on FN BMD (*P* < 0.001). FN BMD was significantly improved by low intensity (*P* < 0.001) and moderate intensity (*P* < 0.001) exercise TH BMD: Significant effect for moderate intensity exercise (*p* < 0.001) whereas low intensity effects were not significant (*p* = 0.31)
Sanchez-Trigo et al. (38) A systematic review and meta-analysis	To explore the effects of non-supervised osteoporosis prevention exercise programs on BMD in pre- and PMW	*Studies included:* 10 *Participants:* 668 (intervention group: *n* = 334; control group: *n* = 334), sample size in studies ranged from 26 to 97 participants *Mean ages:* 38–77 years old *Menopause status:* premenopausal and PMW	*Exercise types:* Impact exercise (jumping or skipping), brisk walking, Tai Chi, unilateral standing, body weight + brisk, walking, and back extension with external loading *Frequency:* Three sessions per week to three sessions per day *Session duration:* 30–60 min *Intensity:* NR *Intervention duration:* 6 months to 12 months	DXA of FN and LS	Non-supervised exercise significantly increased LS BMD (*P* < 0.05) and FN BMD (*P* < 0.05) compared to control DWBHF(dynamic weight-bearing exercise high force, e.g., jogging, jumping, running, dancing, and vibration platform) had a significant positive effect on FN BMD
Mohebbi et al. (39) A systematic review and meta-analysis	To provide a 2022 update regarding the effect of exercise on BMD in the LS, FN, and TH	*Studies included:* 80 studies involving 94 training and 80 control groups *Participants:* 5,581 *Mean ages:* 50–79 years old *Menopause status:* PMW from 0.5 to 24 years	*Exercise type:* 92 groups—aerobic exercise (walking and/or jogging) or combined aerobic and resistance exercise 28 groups—RT Five groups—Tai Chi Six groups—hopping and jumping *Frequency:* 2–9 sessions per week *Session duration:* 10–110 min *Intensity:* Aerobic—60%–80% of MaxHR, RT—70%–80% 1RM *Intervention duration:* 9–18 months	DXA of LS, FN, and TH	Exercise significantly improved BMD of the LS (*p* < 0.001), FN (*p* < 0.001), and TH (*p* < 0.001), using the inverse heterogeneity model (IVhet)
Martyn-St James and Caroll (40) A systematic review and meta-analysis	To evaluate the effects of high-intensity RT on BMD among PMW	*Studies included:* 19, 15 RCTs for meta-analysis *Participants:* 1,164; 578 in treatment group, 586 in control group *Mean ages:* 41–87 years old *Menopause status:* PMW	*Exercise types:* RT: Upper body and lower body *Frequency*: 2–3 days/week *Session duration:* NR *Intensity*: 70%–85% 1RM *Intervention duration*: 6–24 months	DXA of LS, FN, and TH	A significant increase in LS-BMD (*P* = 0.006) following high-intensity RT Results for FN-BMD were inconsistent, with high heterogeneity. There was a non-significant improvement in TH-BMD (*P* = 0.20) following high-intensity RT
Kelley and Kelley (41) A meta-analysis of controlled clinical trials	To examine the efficacy of exercise for improving BMD at the FN in PMW	*Studies included:* 10, 22 groups (12 exercise, 10 control) *Participants:* 595 subjects *Mean ages:* 42–92 years *Menopause status:* PMW	*Exercise types:* 10 groups weight-bearing exercise, two groups non-weight-bearing exercise, and two other groups RT *Frequency*—2–3 days/week *Session duration:* 15–60 min *Intensity*—70%–85% 1RM *Intervention duration:* 8–26 months	DXA of FN	No statistically significant differences within (*P* = 0.429) or between-group (*P* = 0.623) differences in FN BMD
Gonzales-Galvez et al. (42) A systematic review and meta-analysis	To evaluate the effects of RT on physical fitness, physiological variables, and body composition of PMW	*Studies included:* 12; 3 assessed BMD (1997, 2003, and 2012) *Participants:* 198, 116 exercise group, 82 control *Mean age:* 55–65 years old *Menopause status:* PMW	*Exercise type:* RT *Frequency:* 2–3 days (a mean of 2.8 days) *Session duration:* 25–70 min *Intensity:* 50%–90% 1RM *Intervention duration:* average of 5.5 months (range 4–14 months)	DXA site not specified	One of three studies showed a significant improvement in BMD of the LS and TH following RT
Martyn-St James and Caroll (43) A systematic review and meta-analysis	To evaluate the different effects of exercise interventions on hip and spine BMD in PMW	*Studies included:* 15; 10 RCT, 5 non-RCT *Participants:* 1,914, 1,135 treatment group, 779 control *Mean age:* 52–73 years old *Menopause status:* PMW—5.2 median year post	*Exercise type:* High impact, RT, low-impact, and jogging *Frequency:* 3–7 days *Session duration:* NR *Intensity:* NR *Intervention duration:* 9 months—5 years	DXA of LS, FN, and TH	Impact protocols that included jogging mixed with walking and stair climbing (LS-BMD: *p* = 0.02, FN BMD: *p* = 0.001), and protocols that incorporated impact exercise with high-impact RT (LS-BMD: *p* = 0.005, FN-BMD: *p* = 0.03), were effective at both the LS and FN Although heterogeneity was evident in both protocols (*I*^2^ = 88% and *I*^2^ = 73%) for LS-BMD
Howe et al. (44) Systematic review of RCTs	To examine the effectiveness of exercise in preventing bone loss in PMW	*Studies included:* 43 RCTs *Participants:* 4,320 *Mean age:* 45 and 70 years old *Menopause status:* PMW (including those with previous fractures)	*Exercise types:* Static weight bearing (SWB), dynamic weight bearing low force (DWBLF), including walking and Tai Chi. Dynamic weight bearing exercise high force (DWBHF); including jogging, jumping, running, dancing, and vibration platform. Non-weight bearing exercise low force, e.g., low load, high repetition strength training. Non-weight bearing exercise high force, e.g., progressive resisted strength training. Combination (COMB) *Frequency:* –Two to three sessions per week *Session duration:* 45–60 min *Intensity:* 60%–85% 1RM and 80% HRMax *Intervention duration:* 6 months—2.5 years	DXA of FN BMD and LS BMD	The most effective type of exercise intervention on BMD for the FN appears to be NWBHF exercise, such as progressive RT for the lower limbs. The most effective intervention for BMD at the LS was the COMB exercise program (comprising more than one exercise type), with a change of over 3% compared with control groups
Fausto et al. (45) An umbrella systematic review	To analyze the effects of physical exercise on bone health in menopausal women	*Studies included:* 10; 3 SA and 7 MA, published between 2012 and 2020 *Participants:* 6,626 *Menopause status:* pre-menopausal, PeriMW, and PMW	*Exercise type:* Seven studies—aerobic and RT. Two studies—combined RT and aerobic training One study—swimming and two of them with jumping exercises Two studies—Tai Chi *Frequency:* 1–7×/week *Session duration:* 5–60 min *Intensity:* 50%–70% to 45%–80% 1RM *Intervention duration:* 3–24 weeks	DXA scans of FN and LS BMD	A moderate-quality study identified significant benefits from jumping exercise, especially at the hip, with longer sessions of up to 60 min of relatively low intensity. Short-duration and high-intensity aerobic exercises were not effective in improving BMD. Only one study analyzed swimming, obtaining a ‘very low’ classification and high heterogeneity Tai Chi can significantly improve BMD in the LS (46) A study with a “high” level of evidence identified that combined RT and aerobic exercise can preserve LS and FN BMD, whereas RT alone produced only a positive, but non-significant, effect
MHT, exercise and osteoporosis					
Zhao et al. (47) Meta-analysis	To evaluate the combined impact of MHT and exercise on FN and LS BMD in PMW, compared to exercise alone	*Studies included:* 6 *Participants:* 764 *Mean age:* Between 51.8 ± 2.9 and 68.0 ± 3.0 years *Menopause status:* PMW	*Exercise type:* RT (one study); impact exercise (one study—vertical jumps; and mixed loading exercise interventions (four studies), including jumping, skipping, jogging, walking, stair climbing, and RT *Frequency:* 2–6× per week *Session duration:* NR *Intensity:* 50%–80% 1RM, 70% HRMax *Intervention duration:* 12 months *HTR type:* Transdermal estrogen and progesterone, estrogen + MPA, CEO *MHT dosage:* Estrogen 0.625 mg plus MPA 5 mg for 13 consecutive days every third month and CEO 0.625 mg daily *MHT duration:* 1–5.9 years	DXA scans of FN and LS BMD	MHT and exercise generated greater effects on both FN BMD (*p* = 0.039) and LS BMD (*p* = 0.009) than the exercise-only intervention Mixed loading exercise programs were sensitive to MHT in preventing PMW bone loss in the spine (*p* = 0.024)
Born et al. (48) A systematic review and meta-analysis	To determine whether MHT with exercise increases the isolated effect of MHT on bone BMD at the LS and FN	*Studies included:* 6; 1 non-RCT randomized and 5 RCT *Participants:* 774; 219 in the exercise group, 178 in the MHT group, 188 in the MHT + Exercise group, and 189 in the control group. Groups ranged from 8 to 91 participants per group *Mean age:* 52–66 *Menopause status:* PMW	*Exercise type:* High impact, RT, High impact and RT, weight-bearing exercise *Frequency:* 2–6 sessions/week *Session duration:* 10 min to 75 min/session *Intensity:* 60%–80% 1RM, 82% HRMax *Intervention duration:* 11–12 months *MHT Type:* Combined estradiol (2 mg) + norethisterone acetate (1 mg); oral estrogen, transdermal estrogen; oral estrogen and progesterone; transdermal estrogen and progesterone; CEO; estrogen and testosterone; CEO and MPA *MHT dosage:* 26% and 32% oral estrogen; 51% and 61% oral estrogen and progesterone; 12% transdermal estrogen and progesterone; 0.625 mg/day CEO and 5 mg/day MPA; 0.625 mg/day CEO; 8% transdermal estrogen; 5% oral estrogen and testosterone *MHT duration:* 1 year–6 years	DXA FN BMD and LS BMD	MHT plus exercise on LS-BMD was non-significantly (*P* = .27) more pronounced compared with the isolated MHT treatment MHT plus exercise on FN-BMD was non-significantly (*P* = .19) higher than MHT alone
Maddalozzo et al. (49) Case-control trial	To investigate the combined effects of RT (squat and deadlift) performed 2 days per week plus MHT in early PMW	*Participants:* 141 participants (1) Non-MHT plus RT [NMHT plus exercise (*n* = 35)]; (2) MHT plus RT (*n* = 37)]; (3) MHT no RT (*n* = 35)]; (4) control [non-MHT no exercise group (*n* = 34)] *Mean age:* 52.2 *Menopause status:* early PMW	*Exercise type:* Free weight back squat and deadlift exercises *Frequency:* Two sessions week *Session duration:* 50 min supervised *Intensity:* 60%–75% 1RM *Intervention duration:* 12 months *MHT type:* CEO (Premarin®) *MHT dosage:* 0.625 mg/day *MHT duration:* Mean 25 months	DXA scan of FN, TH GT and LS BMD	LS BMD: The control group was significantly lower, *P* ≤ 0.001. MHT plus exercise group resulted in an increase of 0.70 ± 2.2% in LS BMD, while the NMHT plus exercise group had a 0.43 ± 4.3% increase GT BMD: The NMHT plus exercise and MHT plus exercise groups increased BMD 0.43 ± 3.5% and 0.44 ± 2.6%, respectively (*P* > 0.05). The MHT and no exercise group declined −0.60 ± 4.6%, whereas the control group lost −1.5 ± 3.2% FN BMD: NMHT plus exercise and MHT plus exercise groups lost −1.2 ± 4.3% and −0.61 ± 2.9%, respectively. The control group lost the most bone over 1 year at a rate of −3.9 ± 3.8% (*P* > 0.05). The MHT and no exercise group had a similar loss as the NMHT plus exercise group of −1.2 ± 3.3% TH BMD: The NMHT plus exercise and MHT plus exercise groups declined by −0.30 ± 3.1% and −0.52 ± 3.6%, respectively
Bergstrom et al. (50) Randomized pilot study	To investigate the effect of MHT and physical training on BMD in PeriMW	*Participants:* 60 PeriMW, defined as irregular bleeding (6 weeks to 4 months) and/or vasomotor symptoms *Mean age:* 44–51 years old *Menopause status:* PeriMW	*Exercise type:* Walking, RT (arms, legs, back, and stomach), aerobic exercise, and stretching *Frequency:* Walking three times per week, RT and aerobic two times per week *Session duration:* Walking—30 min, RT and aerobic—1 h. *Intensity:* NR *Intervention duration:* 18 months *MHT type:* Estradiol valerate and MPA *MHT dosage:* The MHT group received 2 mg EV daily for 9 days, then 2 mg EV and 10 mg MPA for 12 days, followed by 2 mg EV for 7 days *MHT duration:* 18 months	DXA scan of LS BMD	Both MHT (*p* = 0.006) and physical training (*p* = 0.08) can prevent bone loss in the LS over 18 months in PeriMW, when compared to controls The LS BMD decreased significantly (*p* = 0.0014) in the PeriMW without intervention over a period of 18 months

BMD, bone mineral density; DRT, dynamic resistance training; DXA, dual-energy x-ray absorptiometry; FN, femoral Neck; GRF, ground reaction force; GT, greater trochanter; HRT, hormone replacement therapy; HR, heart rate; LS, lumbar spine; MHT, menopause hormone therapy; NR, not reported; PMW, postmenopausal women; PeriMW, peri-menopausal women; RT, resistance training; RM, repetition maximum; TH, total hip.

### 3.3 Osteoporosis and MHT

Evidence supports the use of MHT in the prevention of osteoporosis. A 25-year cohort study, involving 3,222 women, identified a negative correlation between MHT duration and BMD loss, with greater bone loss in PMW on MHT for 3.75 years compared to 7.66 years (30). Similarly, a 10-year study of 279 PMW examined the effect of continuous combined MHT [Estradiol valerate (E2 V) and MPA] at a low (1 mg E2 V þ 2.5 mg MPA (1 þ 2.5)], medium [1 mg E2 V þ 5 mg MPA (1 þ 5)], and high dose [2 mg E2Vþ5 mg MPA (2 þ 5)] on BMD, and the effect 1-year post-discontinuation after 9 years (31). Long-term low-dose MHT maintained FN BMD for 5–6 years and LS BMD for at least 9 years (31), with high dosages accelerating bone loss after discontinuation.

Combined MHT has been widely studied for its impact on BMD (32–34). The Women’s Health Initiative (WHI) RCT (32) involving 16,608 women with a uterus found a 33% reduction in hip fractures for those on CEO + MPA or CEO compared to placebo, with fracture benefits persisting at 13 years. A meta-analysis (33) involving studies with the number of participants ranging from 24 to 337 showed that combined MHT (predominantly CEO and MPA) had a greater effect on LS BMD than estrogen alone in PMW. Similarly, Ran et al. (34) confirmed that estradiol valerate and MPA increased or maintained BMD in 96 early PMW (aged 40–55 years old).

### 3.4 Osteoporosis and exercise

The impact of exercise on BMD has been widely investigated, with evidence supporting the use of exercise in managing BMD in menopausal women. DXA scans have confirmed the positive effect of exercise on the LS, FN, and TH T-scores (35–40). In contrast, studies have identified that prescribed exercise does not result in changes to TH BMD (35, 40) and the FN (41), though the latter was of lower quality, lacking quality appraisal and sufficient sample sizes.

#### 3.4.1 Exercise types

RT has been extensively investigated, though results have varied. Hejazi et al. (35) identified that RT alone did not significantly increase the BMD of the LS and FN in 2,896 PMW. Gonzales-Galvez et al. (42) support this as two of three studies assessing RT alone had no significant improvement in BMD in 198 PMW, though most studies lasted only 6 months. Whereas RT alone, completed over 6 months, significantly preserved BMD in 1,164 PWM (40), suggesting that the duration of RT is an important factor.

Martyn-St James and Carroli (43) found that combining impact loading (running or jumping) and high-intensity RT with low-impact exercises (stairs and walking) can maintain LS and FN BMD in 1,914 PMW, though study quality was low as blinding of participants is difficult. Further studies (35, 44) have also shown that combined exercise programs significantly improved BMD in the LS, FN, and TH. Kemmler et al. (36) found no significant differences between RT and combined RT on the LS, FN and TH in 2,793 exercising participants; possibly due to participants being 8 years postmenopause, with already low BMD, making it more challenging for interventions to show significant improvements.

#### 3.4.2 Exercise intensity

Studies have investigated the impact of different exercise intensities on BMD. Kistler-Fischbacher et al. (37) found high-intensity exercise more impactful on LS BMD than FN BMD in 3,941 participants, whereas Sanchez-Trigo et al. (38) showed that dynamic high-force weight-bearing exercises (e.g., jogging, jumping, running, and dancing) significantly improved FN BMD, but not LS BMD in 668 participants. Despite these results, both studies only included a small number of high-intensity studies, possibly due to the perceived risk to health; therefore, the between-group analysis may be underpowered (37, 38).

Kistler-Fischbacher et al. (37) identified that FN BMD significantly improved with low-intensity (*P* < 0.001) and moderate-intensity (*P* < 0.001) exercise. Moderate-intensity exercise was most effective for improving TH BMD, though again, there was insufficient data to meta-analyze the impact of high-intensity exercise, reducing the reliability of this conclusion. Evidence on low-intensity Tai Chi is conflicting. Yeh et al. (46) found that Tai Chi significantly improves LS BMD, while Hejazi et al. (35) reported significant increases in FN and TH BMD but not LS. In contrast, Sanchez-Trigo et al. (38) reported no significant effect and Polidoulis et al. (51) a low effect on BMD.

### 3.5 Osteoporosis and MHT and exercise combined

A meta-analysis by Zhao et al. (47) identified that MHT significantly increased the effects of exercise on LS (*p* = 0.009) and FN (*p* = 0.039) BMD compared with exercise alone in 764 PMW. Further analysis supported that combined high-impact activities (jumping, skipping, dancing, and hopping) with high-intensity RT are more responsive to both estrogen-only and combined MHT (47). In contrast, another meta-analysis by Born et al. (48) identified no significant difference between the effect of MHT and exercise vs. MHT alone on the FN and LS in 774 PMW.

Zhao et al. (47) included participants of varying ages, while Born et al. (48) focused on early PMW (within 10 years of menopause). Maddalozzo et al. (49) found that in 141 early PMW (within 3 years), completing only squats and deadlifts was more effective than MHT alone for preserving LS BMD. Bergström et al. (50) found that MHT and exercise, including walking and RT, prevented LS BMD loss in 60 perimenopausal women over 18 months, though a high dropout rate limited long-term conclusions. Thus, these studies suggest that exercise is most effective during the perimenopause and early postmenopause.

## 4 Discussion

Research has highlighted the benefit of using MHT in preventing osteoporosis in menopausal women. MHT prescribed for longer durations resulted in less bone loss (30), with low-dose MHT preferred, as higher doses accelerate bone loss after discontinuation (31). Furthermore, previous research supports the use of combined MHT rather than estrogen alone in preserving BMD (32–34), though this is only applicable in women with a uterus and when progesterone is tolerated. Exercise and MHT combined, compared to exercise alone and MHT alone, significantly improved BMD in menopausal women (47), demonstrating a positive estrogenic response to mechanical loading during exercise on BMD (52). This thus suggests that MHT should be considered as an adjunct to exercise.

None of the included studies explicitly examined the effect of MHT initiation at different time points after menopause, highlighting a gap in the current literature. The timing of MHT initiation post-menopause is a crucial factor in determining its effectiveness in preserving BMD. Research suggests that earlier initiation, typically within the first 10 years of menopause, may provide the greatest skeletal benefits (53). However, our review did not identify consistent data on the impact of delayed MHT initiation. This highlights the need for further research to clarify whether later initiation still offers protective effects or if there is a critical window beyond which benefits diminish.

Despite the effectiveness of MHT in preserving BMD, there are risks associated with its use. The most common estrogen used in these interventions was CEO, administered orally (32, 33, 47, 48), though given the risks reported by the WHI, there has been a significant shift toward transdermal estrogen. Transdermal estrogen, absorbed through the skin via patches, gels, or sprays, has been associated with a lower risk of venous thromboembolism and stroke (54, 55), as it can be prescribed in lower doses as it bypasses the enterohepatic circulation. Furthermore, the most common progesterone used in these studies was MPA (31–34, 47, 48), which, again, has been associated with adverse outcomes. Furthermore, a large meta-analysis by Garthlehner et al. (63), using CEO and MPA, presented similar risks to the WHI, including an increased risk of breast cancer, venous thromboembolism, and stroke when using combined MHT. Consequently, studies that primarily used CEO and MPA may not be fully generalizable to the current population, as prescribing patterns and clinical guidelines have evolved to favor safer delivery methods and formulations.

The International Menopause Society (56) and the British Menopause Society [BMS (57)] advocate the use of MHT as the treatment of choice for osteoporosis prevention; however, this is not supported by further national or international societies (64). The American Association of Clinical Endocrinologists [AACE; (58)] states that in line with FDA licensing guidelines, estrogen should be used for the prevention of postmenopausal osteoporosis in women at significant risk of osteoporosis and when non-estrogen options are unsuitable. The AACE also emphasizes that estrogen should only be prescribed for menopausal symptoms at the lowest dose for the shortest duration (58). Most guidelines prioritize bisphosphonates or denosumab as first-line treatments, recommending MHT only if these are unsuitable, in patients who are under 60 years old, or in those <10 years postmenopause and without previous myocardial infarction, stroke, or breast cancer (2, 59). These guidelines typically evaluate a wide range of evidence, providing strong evidence-based recommendations. Therefore, although MHT can successfully preserve BMD and reduce the risk of osteoporotic fractures, given the risks associated, the initiation of MHT in the prevention of osteoporosis is inconclusive. Further research is required to assess the impact of combined MHT using transdermal estrogen and micronized progesterone on BMD, as these display less harmful characteristics.

This review highlights the importance of exercise in maintaining BMD and managing postmenopausal osteoporosis without hormone supplementation (35–40). Despite these studies displaying heterogeneity between exercise intensity, type, duration, and frequency, the consensus is that PMW should complete combined regimes, including RT and impact activity (also referred to as weight-bearing exercise). To preserve BMD, RT needs to be at an intensity of 70%–85% 1RM completed at least twice per week for over 6 months, with longer interventions likely to produce better results. Impact activity, including jogging and jumping, should be completed at least 3 times per week. Low-impact activity, including Tai Chi, walking, and Pilates, can also be useful in addition to combined training, but should not be used as the sole intervention. Existing guidance from the Bone Health and Osteoporosis Foundation (60) and the National Osteoporosis Guideline Group (61) strongly recommends combined exercise in those at risk of osteoporosis.

Given that the optimal management for preserving BMD in menopausal women involves a combination of MHT and exercise, future research should build on this foundation by examining its broader implications—particularly its impact on mental health. Strong evidence links osteoporosis-related bone loss to psychological distress, including depression, anxiety, and suicidal ideation. A systematic review by Manning et al. (25), published in the *British Journal of General Practice*, found that osteoporosis and fractures, particularly vertebral fractures, are significantly associated with an increased risk of self-harm and suicide. Raising awareness of this dual burden is essential for optimizing patient care. Despite these findings, research has predominantly focused on skeletal outcomes, often relegating mental health to a secondary concern.

Future systematic reviews should therefore prioritize mental health as a primary outcome, investigating the combined effects of MHT and exercise on both skeletal and psychological wellbeing. By adopting this integrated approach, research can provide a more comprehensive understanding of how these interventions contribute to overall wellbeing in menopausal women, ultimately reinforcing the urgency of a comprehensive, multidisciplinary management approach and informing more holistic and effective management strategies.

### 4.1 Strengths and limitations

Although not a systematic review, a comprehensive search strategy was implemented, using the PRISMA-ScR framework. To our knowledge, this is the first review to scope literature from the last 20 years addressing the impact on osteoporosis of MHT, exercise, and MHT and exercise combined. However, we acknowledge that numerous guidelines from international societies and national bodies all consider the information contained in this review as part of their guidance. There is emerging evidence to which this review has added to existing knowledge.

This scoping review has several limitations. First, the inclusion of only systematic reviews and meta-analyses in the exercise and osteoporosis group may introduce bias from lower-quality studies and risk studies being missed. Additionally, the heterogeneity of exercise interventions and MHT preparations across studies, such as varying types, intensities, and durations, makes it difficult to compare outcomes and draw definitive conclusions, reducing the overall reliability and generalizability of the findings. Pharmacological treatments such as bisphosphonates and denosumab were not included, as the aim was to explore non-bisphosphonate strategies. However, we acknowledge this as a limitation, as pharmacological treatments remain essential in osteoporosis management. Future research comparing multiple treatment modalities, including pharmacological approaches, would provide a more comprehensive understanding of osteoporosis management in menopausal women. Finally, the small number of studies included that examined the combined effects on osteoporosis of MHT and exercise (*n* = 4) may impact the generalizability and validity of the results, as a broader body of evidence is needed to draw more robust conclusions about the combined interventions. Furthermore, the variability in study designs, methodologies, and outcome measures may contribute to uncertainty in the overall findings.

## 5 Conclusion

This review highlights that a combination of MHT and structured exercise offers the most effective approach for increasing BMD in menopausal women. For those with a uterus, combined estrogen and progestogen MHT has shown the greatest benefit in preserving bone health. However, due to ongoing debate surrounding the long-term safety of MHT for BMD preservation, exercise remains a critical and universally applicable strategy in the prevention and management of postmenopausal osteoporosis. Specifically, combined RT performed two to three times per week at an intensity of 70%–85% of 1RM, along with impact-loading activities such as jogging, jumping, or hopping at least three times per week, has been shown to be optimal for improving BMD in postmenopausal women. These interventions should be maintained for a minimum of 6 months and progress gradually in intensity and complexity to sustain their effectiveness.

In conclusion, the management of osteoporosis during menopause requires a personalized and multi-faceted approach. While MHT and exercise independently support bone health, their combined use may offer synergistic benefits. Clinical decision-making should weigh individual risk profiles and current evidence to guide effective and safe interventions.

## Data Availability

The original contributions presented in the study are included in the article/Supplementary Material, further inquiries can be directed to the corresponding author.
